# Association of Dietary Patterns with Metabolic Syndrome among Middle-Aged Adults in Shiraz, Iran: Shiraz Heart Study (SHS)

**DOI:** 10.1155/2024/1382031

**Published:** 2024-09-26

**Authors:** Nasrin Motazedian, Mohammad Javad Zibaeenezhad, Mehrab Sayadi, Fatemeh Khademian, Mohaddeseh Hasanzadeh, Ali Ghorbanpour, Ashkan Shamsaeefar

**Affiliations:** ^1^ Transplant Research Center Shiraz University of Medical Sciences, Shiraz, Iran; ^2^ Cardiovascular Research Centre Shiraz University of Medical Sciences, Shiraz, Iran; ^3^ Division of Human Nutrition Department of Agricultural Food and Nutritional Science University of Alberta, Edmonton, AB, Canada; ^4^ Nutritional Health Team (NHT), Shiraz, Iran; ^5^ School of Medicine Shiraz University of Medical Sciences, Shiraz, Iran; ^6^ Faculty of Science York University, Toronto, ON, Canada

## Abstract

**Introduction:**

Metabolic syndrome (MetS) is a noncommunicable disease with a high burden, including the development of type 2 diabetes mellitus, cardiovascular events, and death. It is characterized by abdominal obesity, elevated blood pressure, increased fasting plasma glucose levels, hypertriglyceridemia, and reduced levels of high-density lipoprotein (HDL) cholesterol. MetS is preventable by modifying lifestyle and dietary patterns, which are major contributing factors. This research aimed to investigate the dietary patterns of the Shiraz Heart Study (SHS) and their associations with the occurrence of MetS and its components among middle-aged residents of Shiraz.

**Methods:**

Based on data from the Shiraz Heart Study (SHS), a prospective cohort study, the nutritional status of 1,675 participants was assessed using a food frequency questionnaire (FFQ). Three food patterns were extracted from the analysis named as vegan, western, and carbohydrate. Subjects were categorized into three levels for three major dietary patterns: low, moderate, and high, based on their adherence to each pattern. After adjusting the effect of co-founder variables, the relationship between dietary patterns, and the risk of developing MetS was analyzed.

**Results:**

Of the 1,675 participants, 728 (43.5%) of them were male. The prevalence of MetS was 47.2%. Multivariate logistic regression analysis showed that high adherence to the vegan pattern was negatively associated with the occurrence of MetS (*P* value <0.001), while low adherence to the western pattern was also negatively associated (*P* value <0.05).

**Conclusion:**

Healthier diets, such as vegan diets, are significantly related to lower rates of MetS among the 40–70-year-old people in Shiraz, Iran.

## 1. Introduction

Metabolic syndrome (MetS) is a growing concern worldwide, which exposes people to serious cardiovascular risks; further, it is the main cause of death in Iran. The prevalence of MetS among Iranians was 30.4%. The total number of deaths caused by metabolic risk factors has raised from 1990 to 2019. High SBP was the leading risk factor regarding attributed age-standardized death rates reaching 157.8 per 100,000 person-years, in 2019 [1, 2]. MetS is characterized by the concurrent presence of at least three out of five specific symptoms: abdominal obesity, elevated blood pressure, increased fasting plasma glucose levels, hypertriglyceridemia, and reduced levels of high-density lipoprotein (HDL) cholesterol [3]. In recent decades, the prevalence of MetS has increased dramatically, most likely due to alterations in lifestyle, socio-economic status, and eating habits [4, 5].

Individuals with MetS are at risk of developing type 2 diabetes and cardiovascular diseases, as mentioned above [6]. Regarding the risk factors of cardiovascular disease, emphasizing lifestyle changes, as the primary strategy, is crucial in preventing and managing MetS [7]. One of the major challenges in the management of MetS is addressing the risks related to modifiable factors via lifestyle interventions, such as obesity, physical activity, and diet [4].

Aligned with existing research, there is a well-established relation between the intake of particular food groups or essential nutrients and the propensity for MetS to manifest [8]. Notably, the prevalence of MetS has been significantly influenced by dietary patterns, highlighting their significant impact on this context. The components of MetS were positively associated with a meat—instant food dietary pattern (rich in animal protein, saturated fat, sweets, sodium, and food additives). Meanwhile, the components of MetS were inversely associated with veg-seafood dietary pattern (rich in dietary fiber, vitamins, minerals, and unsaturated fat) or a cereal—dairy dietary pattern (rich in dietary fiber, antioxidants, phytochemicals, complex carbohydrates, prebiotics, and probiotics) [9]. Dietary pattern analysis discerns consumption patterns of nutrients rather than single food items [6]; hence, it is frequently considered a more efficient approach to mitigating the onset of diseases compared to the singular consumption of foods [9]. The impact of vitamin D supplementation on components of metabolic syndrome has been reported [10]. A comprehensive investigation indicated that adherence to a Mediterranean diet, characterized by an abundance of vegetables, fruits, whole grains, cereals, and seafood, correlated concomitantly with a reduced incidence of MetS and a consequential decline in mortality attributable to this syndrome [11]. Conversely, a “western” dietary pattern, marked by heightened consumption of red and processed meats, refined grains, alcoholic beverages, and fried cuisine, increased the susceptibility to MetS [4].

Thus, the primary prevention of MetS is important to avoid severe consequences [6]. Despite the increase in MetS risk factors, the relationship between dietary patterns and MetS in Shiraz remains uncharted territory. This research aimed to determine the primary dietary patterns of the Shiraz Heart Study (SHS) as well as their associations with the incidence of MetS and its constituent components among middle-aged adults residing in Shiraz, a densely populated urban center in Iran.

## 2. Methods and Materials

This cross-sectional study was designed based on the available data from the SHS [12], a prospective cohort study targeting the general population of Shiraz aged 40–70 years, with the primary aim of assessing the risk of cardiovascular diseases over 10 years. In this study, the participants' nutritional status was evaluated, using a food frequency questionnaire (FFQ). Metabolic syndrome (MetS) was determined based on the Adult Treatment Panel III (ATP III) definition, which classifies individuals as having MetS if they exhibit at least three of the following five risk factors:

1. Abdominal obesity, defined as an abdominal circumference of ≥88 cm for women and ≥102 cm for men.
2. High triglycerides, defined as ≥150 mg/dl or under specific treatment.
3. Low HDL cholesterol, defined as <40 mg/dl for men and <50 mg/dl for women, or under specific treatment.
4. High blood pressure, defined as systolic/diastolic values of ≥130/85 mmHg, or under specific treatment.
5. Fasting blood sugar (FBS) ≥100 mg/dl or diagnosed diabetes.

There are several methods for modeling food consumption. In this study, we used exploratory factor analysis (EFA) [13, 14]. We defined 35 food groups to derive the dietary patterns. The number of desirable factors was extracted using the principal component method and selected by considering the scree plot and explained variance. For better interpretability, Varimax rotation was applied. Three food patterns were extracted from the analysis named as vegan, western, and carbohydrate pattern. The score of each subject in each food pattern was calculated based on every factor and then standardized. Finally, subjects in each food group were divided into three groups based on the tertile of factor's score. In each of the three food patterns, the group in the first third of the score was classified as the group that used that food pattern the least (Low group). The second group used that pattern at an average level (Moderate group), and the third group used each food pattern the most (High group).

### 2.1. Inclusion and Exclusion Criteria

Following the main cohort study, the participants were included if they were between 40 and 70 years old. The subjects were excluded if they had any severe diseases.

### 2.2. Data Analysis

Before analyzing the data, normality was assessed using skewness, kurtosis, Q-Q plots, and P-P plots. Outliers were removed, resulting in a final sample of 1,675 participants with complete micronutrient information. Quantitative data are presented as mean ± SD and qualitative data as number (%). An independent sample *t*-test was used to compare the age of participants with and without MetS, while a one-way analysis of variance (ANOVA) was used to compare the age across the three levels of adherence to dietary patterns. The chi-square test was employed to compare the categorical data between the groups. Also, we used the chi-square test to compare the categorical data between groups. Unconditional multiple logistic regression analysis was conducted to investigate the relationship between MetS and dietary patterns, adjusting for confounding variables such as age, gender, smoking, and marital status. All tests were two-sided with a significance level of 5%. Data analysis was performed using SPSS software for Windows, version 16.0 (Chicago, SPSS Inc.).

## 3. Results

Out of 1,675 participants, 728 (43.5%) were men, with a mean age of 53.39 ± 8.28 years. Among them, 342 were smokers, and the majority (*n* = 1,564, 90.5%) were married. The prevalence of MetS was 47.2%. A comparison of the demographic characteristics between participants with and without MetS is shown in Table 1. Participants with MetS were older (*P* < 0.001), and the prevalence of MetS was higher in women than in men (*P* < 0.001). However, there was no statistically significant difference in the prevalence of MetS between smokers and nonsmokers (*P*=0.743), nor between married and nonmarried participants (*P*=0.403).

Table 2 summarizes the demographic characteristics according to the participants' adherence to three different dietary patterns. Participants who adhered more closely to the vegan and high-carbohydrate patterns were older, while those following the western pattern were younger (*P* < 0.05). The distributions of gender, smoking, and marital status were statistically similar across the three levels of adherence to the vegan diet (*P* > 0.05). Men and smokers were less likely to adhere to the western and high-carbohydrate patterns compared to women and nonsmokers (*P* < 0.001). Moreover, married people were less likely to follow these two patterns compared to nonmarried individuals (*P*=0.016).

The prevalence of MetS based on three patterns is depicted in Table 3. Higher levels of triglycerides, blood pressure, and lower HDL were more common in participants with lower adherence to the vegan pattern (*P* < 0.05). The prevalence of MetS was also higher in those with low adherence to the vegan pattern (*P* < 0.001).

Conversely, higher waist circumference, FBS, and lower HDL were more common among those who followed the western pattern more closely (*P* < 0.05). The prevalence of MetS was also higher among these participants (*P* < 0.001). Similarly, high waist circumference and FBS were more common among those with greater adherence to the high-carbohydrate pattern (*P* < 0.05), and the prevalence of MetS was higher in this group as well (*P* < 0.001).

The results of the multivariate logistic regression model (Table 4) indicated that MetS was related to the vegan and western dietary patterns. Specifically, lower adherence to the vegan pattern increased the chance of MetS (OR = 1.36, 95% CI: 1.06–1.74, *P*=0.015 for moderate level and OR = 1.62, 95% CI: 1.26–2.08, *P* < 0.001 for low level). On the other hand, higher adherence to the western pattern also increased the chance of MetS (OR = 0.75, 95% CI: 0.58–0.96, *P*=0.024 for moderate level).

## 4. Discussion

One of the significant challenges in public health today is metabolic syndrome (MetS). The rising prevalence of MetS can be partly attributed to the aging population and lifestyle factors such as diet [4, 8]. The role of diet in health is well established. Healthy and balanced dietary patterns can help people achieve and maintain an appropriate body weight and provide the necessary energy to perform tasks effectively [15].

This study compares the association of major dietary patterns with MetS and its components in a populous city in Iran, finding the prevalence of MetS to be 47.2%. MetS was more prevalent among older adults, women, and smokers, while marital status did not show a statistically significant difference.

A meta-analysis of 69 studies found the overall prevalence of MetS among the Iranian population to be 30.4%, with a higher prevalence among women and the elderly [16], which aligns with our results. Other studies have reported lower prevalence rates of MetS, compared to our study. Some studies indicated a higher prevalence of MetS among older adults and men [4, 17, 18]. Additionally, MetS was more common among individuals with lower education levels, married participants, unemployed individuals, and smokers [4]. While researchers have reported that the prevalence of MetS increases with age, the findings regarding gender have been mixed. This variation may be due to the different age groups studied. For example, Cho Y-A et al. reported a lower prevalence of MetS among women, but the prevalence was equal among both genders after the age of 60 years. Differences in activity levels due to jobs and dietary patterns may also influence these results, suggesting a need for further studies to investigate the impact of dietary patterns on MetS separately for each gender [19].

We used a Food Frequency Questionnaire (FFQ) to compare the dietary patterns of the participants. The results of the multivariate logistic regression model showed that MetS was related to vegan and western dietary patterns. The westernized pattern is characterized by the consumption of meat and its products, fried dishes, and sugar-sweetened beverages [17]. Woo et al. reported that MetS was more prevalent among men whose diet mainly consisted of meat [18]. Previous studies have shown that adherence to vegan/Mediterranean diets is significantly associated with a reduced risk of MetS, while adherence to Meat/western patterns is related to an increased risk [8, 20–24]. Fiber-dominant diets reduce visceral and subfascial fat in muscle tissues, promoting glucose homeostasis and reducing the likelihood of developing MetS [21, 25, 26].

The association of carbohydrate pattern with MetS has been distinguished in studies [27, 28]. Among the Asian population, a stronger relationship between carbohydrate intake and the risk of MetS was noted compared to the non-Asian population, possibly due to differences in the amount of carbohydrates consumed [19]. This study found a significant relationship between adherence to a carbohydrate-rich diet and MetS and its components, including waist circumference, FBS. Liu et al. reported a positive association between carbohydrate intake and MetS in a meta-analysis study [28]. As muscle insulin resistance increases with age, postprandial muscle glycogen synthesis decreases, leading to a shift from carbohydrate to lipid synthesis in the liver and resulting in hypertriglyceridemia [17].

We also found notable relationships between MetS and adherence to vegan and western diets, but no significant association with the carbohydrate diet. Nevertheless, the three dietary patterns were related to some components of MetS, depending on the type of diet. In this study, higher blood pressure, TG, and low HDL were related to low adherence to the vegan diet. Montemayor et al. reported that high adherence to the Mediterranean diet resulted in significant declines in BMI, body weight, waist circumference, systolic BP, and diastolic BP [23]. This is likely due to the lack of animal-originated fat and the low glycemic index of the diet. The vegan diet adjusts lipid metabolism, especially TG [21].

One strategy for preventing MetS is lifestyle modification, including increased physical activity, adherence to a healthy diet, and dietary diversity [29, 30].

### 4.1. Limitations

This is a cross-sectional study, so the causal link between dietary patterns and the development of MetS remains unclear. Recall bias is another inevitable limitation that may have influenced the results. Nonetheless, the study benefits from a large sample size, investigating dietary patterns and metabolic syndrome in Shiraz, Iran.

## 5. Conclusion

About half of the population met the criteria for MetS, with a higher prevalence in older adults and females. Multivariate logistic regression analysis revealed a significant relationship between MetS and high adherence to two dietary patterns: vegan and western. Specifically, those following a western diet, which includes higher amounts of meat, are more prone to developing MetS. In contrast, individuals with a vegan diet tend to experience less MetS over their lifetime. Adopting a vegan diet is recommended to help reduce the risk of MetS.

## Figures and Tables

**Table 1:** Comparison of demographic characteristics in participant with and without MetS.

Variables	Total	MetS		*P* value
		Yes (*n* = 790)	No (*n* = 885)	
Age	53.39 ± 8.28	54.86 ± 8.07	52.08 ± 8.25	<0.001
Gender				<0.001
Male	728 (43.5)	275 (37.8)	453 (62.2)	
Female	947 (56.5)	515 (54.4)	432 (45.6)	
Smoking				0.743
Yes	342 (20.4)	164 (48.0)	178 (52.0)	
No	1333 (79.6)	626 (47.0)	707 (53.0)	
Marital status				0.403
Married	1516 (90.5)	710 (80)	809 (79)	
Other	159 (9.5)	79 (8.9)	80 (10.1)	

Data were presented as mean ± SD and number (%) for continuous and categorical data, respectively. Mets = metabolic syndrome.

**Table 2:** Comparison of demographic characteristics in the participants with 3 levels of adherence to dietary patterns.

Variables	Vegan pattern			*P* value
	High	Moderate	Low	
Number	562	554	559	
Age	55.13 ± 8.08	53.53 ± 8.23	51.50 ± 8.14	<0.001
Male gender	228 (40.6)	255 (46.0)	245 (43.8)	0.180
Smoking	117 (20.8)	119 (21.5)	106 (19.0)	0.557
Married (yes/other)	508 (90.4)	495 (89.4)	513 (91.8)	0.385

	Western pattern			
	High	Moderate	Low	

Number	557	562	556	
Age	52.71 ± 8.37	53.51 ± 8.36	53.96 ± 8.07	0.039
Male gender	182 (32.7)	242 (43.1)	304 (54.7)	<0.001
Smoking	102 (18.3)	95 (16.9)	145 (26.1)	<0.001
Married (yes/other)	491 (88.2)	518 (92.2)	507 (91.7)	0.058

	High-carbohydrate pattern			
	High	Moderate	Low	

Number	559	563	553	
Age	54.99 ± 8.15	53.26 ± 8.22	51.91 ± 8.21	<0.001
Male gender	185 (33.1)	229 (40.7)	314 (56.8)	<0.001
Smoking	66 (11.8)	104 (18.5)	172 (31.1)	<0.001
Married (yes/other)	494 (88.4)	506 (89.9)	516 (93.3)	0.016

Data are presented as mean ± SD and number (%) for continuous and categorical data, respectively.

**Table 3:** Frequency distribution of MetS and its components according to different food patterns.

Variables	Vegan pattern			*P* value
	High	Moderate	Low	
High waist circumference	351 (62.5)	335 (60.5)	364 (65.1)	0.274
High TG or on antilipid drug	282 (50.2)	314 (56.7)	326 (58.3)	0.015
High FBS or diabetic	147 (26.2)	165 (29.8)	175 (31.3)	0.149
High blood pressure or on drug	251 (44.7)	256 (46.2)	297 (53.1)	0.010
Low HDL	235 (41.8)	270 (48.7)	266 (47.6)	0.045
MetS	233 (41.5)	263 (47.5)	294 (52.6)	0.001

	Western pattern			
	High	Moderate	Low	

High waist circumference	380 (68.2)	349 (62.1)	321 (57.7)	0.001
High TG or on antilipid drug	323 (58.0)	293 (52.1)	306 (55.0)	0.114
High FBS or diabetic	202 (36.3)	158 (28.1)	127 (22.8)	<0.001
High blood pressure or on drug	273 (49.0)	279 (49.6)	252 (45.3)	0.296
Low HDL	293 (52.6)	247 (44.0)	231 (41.5)	0.001
MetS	307 (55.1)	252 (44.8)	231 (41.5)	<0.001

	High-carbohydrate pattern			
	High	Moderate	Low	

High waist circumference	377 (67.4)	367 (65.2)	306 (55.3)	<0.001
High TG or on antilipid drug	317 (56.7)	323 (57.4)	282 (51.0)	0.063
High FBS or diabetic	175 (31.3)	177 (31.4)	135 (24.4)	0.013
High blood pressure or on drug	269 (48.1)	274 (48.7)	261 (47.2)	0.884
Low HDL	255 (45.6)	260 (46.2)	256 (46.3)	0.971
MetS	270 (48.3)	283 (50.3)	237 (42.9)	0.037

Data are presented as mean ± SD and number (%) for continuous and categorical data, respectively. Mets = metabolic syndrome, TG = triglyceride, FBS = fasting blood sugar, HDL = high-density lipoprotein.

**Table 4:** The effect of different food patterns on MetS using multiple logistic regression analysis.

Variables	OR (95% CI)			*P* value
	OR	Lower bound	Upper bound	
Age	1.05	1.03	1.06	<0.001
Gender (male/female)	0.40	0.32	0.51	<0.001
Smoking (yes/no)	1.36	1.05	1.76	0.019
Married status (married/other)	0.117	1.32	0.93	1.88
Vegan pattern				
High	Reference			
Moderate	1.36	1.06	1.74	0.015
Low	1.62	1.26	2.08	<0.001
Western pattern				
High	Reference			
Moderate	0.75	0.58	0.96	0.024
Low	0.78	0.61	1.01	0.069
High-carbohydrate pattern				
High	Reference			
Moderate	1.25	0.97	1.60	0.076
Low	1.05	0.81	1.36	0.706

## Data Availability

The datasets analyzed during the current study are available from the corresponding author upon request.
